# Control Measures Used during Lymphogranuloma Venereum Outbreak, Europe

**DOI:** 10.3201/eid1404.061583

**Published:** 2008-04

**Authors:** Aura Timen, Marlies E.J.L. Hulscher, Dieuwke Vos, Marita J.W. van de Laar, Kevin A. Fenton, Jim E. van Steenbergen, Jos W.M. van der Meer, Richard P.T.M. Grol

**Affiliations:** * National Institute for Public Health and the Environment (RIVM), Bilthoven, the Netherlands; † Dutch Association for Public Health Services, Utrecht, the Netherlands; ‡ University Medical Centre, St. Radboud, Nijmegen, the Netherlands; § European Centre for Disease Prevention and Control, Stockholm, Sweden; ¶ Health Protection Agency, London, UK

**Keywords:** Lymphogranuloma venereum, outbreaks, response, alert, quality, research

## Abstract

To assess the response to the reemergence of lymphogranuloma venereum, we conducted a cross-sectional survey by administering a structured questionnaire to representatives from 26 European countries. Responses were received from 18 countries. The ability to respond quickly and the measures used for outbreak detection and control varied. Evidence-based criteria were not consistently used to develop recommendations. We did not develop criteria to determine the effectiveness of the recommendations. The degree of preparedness for an unexpected outbreak, as well as the ability of countries to respond quickly to alerts, varied, which indicates weaknesses in the ability to control an outbreak. More guidance is needed to implement and evaluate control measures used during international outbreaks.

## Abstract 2

The degree of preparedness may affect the ability to respond quickly and to control an outbreak.

Responding effectively to international communicable disease emergencies is a complex process that involves national and international cooperation. Efforts should be aimed at managing patient care and containing the disease by interrupting the chain of transmission (*1*,*2*). The severe acute respiratory syndrome outbreak has shown the need for being prepared and being able to deal with international emergencies in a consistent way; all countries need to be prepared and able to respond to an outbreak. Countries throughout Europe have developed preparedness plans to face a possible pandemic caused by a new influenza virus. But even with a well-acknowledged threat like an influenza pandemic, differences in preparedness between countries exist (*3*,*4*). The differences might be even greater when timely control measures are needed for outbreaks that remain unnoticed for a long time.

Systems for surveillance and outbreak management among European countries differ, as do their health policies and guidelines. We wondered whether these differences could lead to different outbreak control measures and therefore to differences in the effectiveness of these control measures. We studied the quality and timeliness of public health actions during the reemergence of lymphogranuloma venereum (LGV) among men who have sex with men (MSM) in Europe from January 2004 to February 2006. In January 2004, the European Surveillance of Sexually Transmitted Infections Network (ESSTI) issued an international alert. This action was considered a trigger for countries to identify possible cases; define, inform, and investigate the population at risk; and to implement control measures. The resurgence of LGV in Europe contained many features similar to an infectious disease emergency: it occurred unexpectedly; there was delay in the recognition of cases, which allowed the disease to spread within the risk group; and there was no preconceived outbreak control plan. Moreover, in many countries, LGV is not reportable and surveillance is voluntary.

Our study of the response to this LGV outbreak demonstrates the need for a unified response to new, unexpected, infectious diseases. We assessed the similarities and differences in how various countries managed the LGV outbreak to identify common practices and to formulate criteria for improving the response to international outbreaks.

## Participants and Methods

A cross-sectional survey was conducted from October 2005 through February 2006 among the countries participating in ESSTI and in Switzerland. The ESSTI then consisted of 22 member states of the European Union plus Iceland, Norway, and Turkey. Scotland was included as an individual respondent and participated in the network as such. In collaboration with the ESSTI steering group, we developed a structured questionnaire and sent it to each country’s representative (surveillance leads and reference microbiologists).

The items on the questionnaire were based on a framework derived from the literature about outbreak management (*1*,*3**–**5*). In addition, to assess the quality of the development and implementation of key recommendations for controlling the outbreak, we used parts of the international AGREE instrument (www.agreecollaboration.org) for appraising guidelines and guideline development programs.

The questionnaire was divided into 4 sections. The first section was dedicated to the alert and initial response to LGV and included 8 questions about actions taken after the ESSTI alert, risk assessment, and occurrence of cases. The second section included 8 questions about the development of outbreak control measures and gathered information about how evidence was collected and analyzed, how measures were formulated, when experts were consulted, and how recommendations were updated. The third section included 9 questions about the content of outbreak control measures (i.e., case identification, case definitions, laboratory confirmation, treatment, reporting, and interventions for health professionals and the groups at risk). The fourth section addressed implementing outbreak control measures (i.e., strategies, dissemination of information, targets for monitoring effectiveness, and additional resources).

Questionnaires were completed electronically or on paper, and data were analyzed by SPSS 12.0 (Chicago, IL, USA). LGV is a sexually transmitted infection (STI) caused by *Chlamydia trachomatis* serovars L1, L2, and L3. Contrary to infection with other serovars, infections with *C. trachomatis* L1–3 are not limited to the mucosa but rather are often invasive and can spread to the lymph nodes, which results in a more severe clinical outlook. In industrialized countries, cases are incidentally imported from tropical and subtropical areas where the disease is endemic (*6*). Public health measures are usually restricted to contact tracing and adequate management of sex partners in individual cases; outbreak management is not needed. By the end of 2003, 13 cases had been reported to the public health authorities in the Netherlands, followed by a substantial increase in cases in subsequent months. The cases were seen among MSM. Clinical signs were mostly gastrointestinal and included proctitis, purulent or mucous anal discharge, and constipation (*7*). In the early days of the outbreak, the potential for international spread was recognized because patients reported having had sexual contacts in other countries such as Belgium, the United Kingdom, and France (*8*).

To create awareness, a message was sent through the Early Warning and Reporting System of the European Union and through the ESSTI. Since then, LGV cases have been identified in several European countries, the United States (*9*), and Canada (*10*). Most patients were HIV positive (*11*), and some were hepatitis C positive (*12*).

## Results

The questionnaire was sent to 26 countries; 11 of these countries had reported outbreaks of LGV in the past. Completed questionnaires were received from 18 countries (Austria, Belgium, Denmark, Finland, France, Germany, Ireland, Italy, the Netherlands, Norway, Portugal, Scotland, Slovenia, Spain, Sweden, Switzerland, United Kingdom, and Turkey). Of the 18 questionnaires, 12 were completed by medical doctors, 4 by medical epidemiologists, and 2 by researchers/microbiologists. In 5 countries (Belgium, Ireland, Portugal, Slovenia, and Sweden), the questionnaire was filled in by 2 or more experts from different areas of expertise. The 8 countries that did not respond to either the questionnaire or the electronic reminders (Slovak Republic, Poland, Malta, Latvia, Iceland, Cyprus, Estonia, and Greece) were excluded from the analysis. A complete overview of the activities reported for controlling LGV and their development and implementation is given in the (Tables 1 and 2).

**Table 1:** Control measures used by 18 European countries during an LGV outbreak*

Category	All countries (n = 18)	Countries with cases (n = 11)
Initial alert and response		
Alert and response issued	11	9
Enhanced surveillance	8	7
Voluntary reporting	9	7
Provisional guidelines	9	7
Information dissemination	11	9
Educational activities	6	6
Development of control measures		
Outbreak management team (advisory team)	5	5
Initial risk assessment performed	8	7
Methods to collect evidence		
Hand search literature	11	9
Search electronic databases	10	7
Search patient entry data	7	6
Search unpublished data	4	4
Methods to analyze evidence		
Decision analysis	5	3
Meta-analysis	0	0
Nonsystematic review	5	4
Systematic review	4	4
Experience based	8	7
Methods to formulate measures		
Subjective review	8	6
Informal expert consensus	10	8
Formal expert consensus	4	4
Evidence based	0	0
Procedure for updating key recommendations	11	4
National LGV guideline	4	3
Formal authorization process of the guideline	2	2
Content of control measures		
Active case finding	9	7
Contact tracing	7	6
Partner notification	5	4
Screening risk group	5	3
Activities targeting risk groups	10	8
Alerting general public	1	1
Alerting general practitioners	8	5
Alerting STI clinics	11	9
Alerting public health physicians	9	9
Alerting microbiologists	9	6
Alerting hospitals	5	3
Alerting GUM and gastroenterologists	8	6
Alerting HIV specialists	3	3
LGV notifiable†	5	4
National case-register for LGV†	9	7

*Data gathered through a survey. LGV, lymphogranuloma venereum; STI, sexually transmitted infection; GUM, genitourinary specialists. †Data were missing for 3 countries for this category.

**Table 2:** Implementation of control measures used by 18 European countries during an LGV outbreak*

Implementation measure	All countries (n = 18)	Countries with cases (n = 11)
Disseminating educational materials	9	8
Conferences for professionals	3	3
National bulletins	5	4
Outreach visits	0	0
Computer reminders	0	0
Changes in medical records systems	0	0
Changes in facilities and equipment	0	0
Additional finances	3	3
Strategy for media communication	6	5
Involvement of MSM society in dissemination of information	11	8
Targets to monitor effectiveness	0	0

*Data gathered through a survey. LGV, lymphogranuloma venereum; STI, sexually transmitted infection; MSM, men who have sex with men. †Data were missing for 3 countries for this category.

### Initial Alert and Response

After the ESSTI alert in January 2004, timely national alert and response systems were set up by 11 of the 18 responding countries. These systems included provisional control guidelines (9 countries), voluntary reporting (9 countries), and tools for disseminating information to health professionals (11 countries). Of the 11 countries who undertook early alert and response activities, 9 also reported cases. The main objectives of the alert were active case finding (11 countries), assessing the size and nature of the outbreak (10 countries), and providing appropriate clinical care (9 countries). In 4 countries, the initial alert and response were undertaken by professionals from the STI surveillance system in collaboration with specialists in outbreak control. In the other 7 countries, only surveillance specialists were involved.

### Development of Outbreak Control Measures

Five countries had a national outbreak management team or advisory committee that provided scientific advice on surveillance and outbreak management. The multidisciplinary outbreak management teams always included epidemiologists and microbiologists; less frequently included were molecular biologists, dermatovenereologists, genitourinary specialists, and communicable disease control specialists. In 1 country, communication experts and social scientists also participated in the outbreak management teams. No general practitioners, nurses, patients, or policymakers were involved in outbreak management teams. Of the 18 countries, control measures were aimed primarily at identifying new cases (8 countries) and promoting awareness among the risk group (10 countries) and STI clinics (11 countries). A risk assessment was performed by 8 countries.

When developing recommendations for outbreak control, criteria varied with the 18 countries (Tables 1 and 2). Evidence was systematically collected by literature (11 countries) and electronic database searches (10 countries). Informal consensus procedures were mostly used to formulate recommendations (10 countries) based on experience-based analysis of evidence (8 countries). Procedures for updating control measures were available in 11 countries. A total of 4 countries developed national, multidisciplinary guidelines for LGV control, 2 of which issued authorization procedures for the guidelines.

### Content of Outbreak Control Measures

Active case finding was initiated by 9 countries and contact tracing by 7. Five countries implemented both. Information activities for the group at risk were performed by 10 countries and 11 countries alerted their STI clinics. An overview of all the control measures is given in the (Tables 1 and 2). A total of 11 respondents (Denmark, Germany, Norway, Sweden, Spain, United Kingdom, Scotland, Austria, the Netherlands, Ireland, and Belgium) used an identical case definition for confirmed cases: MSM or sexual contacts of patients with LGV, who had anorectal or inguinal syndrome and positive nucleic acid amplification tests (NAAT) for *Chlamydia trachomatis* genotype L1, L2, or L3. From these 8 countries, case definitions were also issued for probable and possible cases and differed widely according to laboratory criteria.

Laboratory diagnosis of *C. trachomatis* was performed by NAAT on the following samples: rectal swabs (12 countries), biopsy material from lesions (8 countries), urethral swabs (5 countries), and urine (2 countries). Genotyping to confirm the presence of serovars L1–L3 was also available from 11 countries. Supplementary testing of patients for concurrent STIs was recommended as follows: HIV (8 countries), syphilis (5 countries), hepatitis C (3 countries), hepatitis B (3 countries), and *Neisseria gonorrhoeae* (2 countries).

With respect to antimicrobial therapy, various regimens and different doses were used. For 9 countries doxycycline (100 mg 2×/day for 21 days) was the first choice of treatment. Alternatives mentioned were tetracycline (2 g/day), minocycline (300 mg loading dose followed by 200 mg 2×/day), and erythromycin (500 mg 4×/day). Clinical and laboratory follow-up of the patients was performed by 10 countries.

### Implementation of Outbreak Control Measures

The control measures were implemented by disseminating educational materials in 9 countries, disseminating national bulletins in 5, and holding meetings and conferences for professionals in 3 countries. Most countries (11/18) had the risk group help disseminate information. Targets to monitor the effectiveness of recommendations were not formulated by any country.

## Discussion

This outbreak of LGV had special features with high clinical and public health significance. First, recognition of cases was difficult due to the unusual clinical presentation that mimics inflammatory bowel disease. Second, the diagnosis of LGV involved invasive procedures for collecting samples and required NAAT, which were not licensed for rectal specimens. Furthermore, patients mostly belonged to sex networks of MSM in large cities with numerous anonymous partners from different countries (*13*) and where (international) contact tracing was difficult. In most European countries, LGV is not notifiable by law so cases are likely to be dealt with outside the public health domain. The potential of unnoticed further spread and the risk for simultaneous transmission of other infections, such as HIV and syphilis, increased the public health importance of this outbreak.

Differences were seen between countries with respect to ability to rapidly respond and implement measures that are needed to detect or to control a possible outbreak. Countries that reported cases of LGV were more likely to recommend control measures although measures were also needed to detect possible cases. To identify and diagnose cases, clinical specialists and public health physicians, as well as the risk group, must be aware of the outbreak, particularly for an LGV outbreak. LGV is a rare disease in Europe, and often healthcare workers are not aware of the clinical features of the disease.

Outbreak control measures require collaboration between persons in multiple specialties, such as specialists in surveillance, communicable disease control, health promotion, and physicians involved in the direct patient care (venereologists, genitourinary medicine specialists, gastroenterologists, microbiologists) that do not necessarily work together in other circumstances. In this outbreak, information from the surveillance systems was as important for health providers as for policymakers; this information had to lead to immediate recognition of a public health threat and direct action.

However, in the management of LGV patients, differences were seen between countries with respect to case definitions, laboratory testing, and antimicrobial drug treatment. With most patients belonging to international sex networks, uniform diagnostic procedures and treatment protocols would have been helpful for ensuring a uniform approach to outbreak control. Furthermore, control measures were impaired because in many countries LGV is not a notifiable disease; therefore, there is no legal basis for disclosing names of sexual contacts to facilitate contact tracing and prevent further spread. Contact tracing was made even more difficult because of the numerous anonymous sexual contacts in various European cities.

Criteria for evidence-based development of recommendations were not always consistently used to extract and analyze evidence for best practices during the LGV outbreak, which led to differences in outbreak management. Specific targets for monitoring the effectiveness of recommendations were not formulated by any country. One strong point was the acknowledgment by many countries of the importance of having the risk group, MSM, disseminate alerts and advocate awareness.

Until now, the reemergence of LGV has affected MSM in 11 European countries. The ESSTI alert prompted these countries to take action to identify cases early, improve the management of cases, and assess the size of the outbreak. Of the 18 respondents, 7 stated that they had not taken any action at this stage for various reasons: they did not receive the alert (Turkey, Slovenia) or they did not participate in the ESSTI (Switzerland). Coordination at the European level should encourage and monitor the response of all countries to alerts.

Our study has some limitations. We assumed that all countries that were participating in the ESSTI network in 2005 had also been informed about the LGV outbreak. Later, it became clear that the countries that had joined the European Union on May 1, 2004, did not receive the ESSTI alert. Because only 1 of these new European Union member countries completed the questionnaire, it was also impossible to assess how outbreak control measures were developed and implemented. Another limitation inherent to the method used was that not all key persons involved in the control of LGV were able to fill out the questionnaire. As the questionnaire was sent to the country representatives in the ESSTI, it is possible that not all relevant information was available on the control measures and activities that had taken place at regional or local levels. Furthermore, the quality of the outbreak management process and the development of outbreak measures could only be assessed indirectly on the basis of the answers to the questionnaire because only a few countries provided more detailed documents like guidelines or articles. The LGV outbreak is still ongoing in Europe, and since the completion of this study more countries may have undertaken measures to identify and treat cases and to prevent further transmission.

Our findings are helpful for understanding the responses to unexpected disease outbreaks. However, we do acknowledge that LGV is an STI (rather than a quick-spreading communicable, airborne disease) and therefore, affects a minority of sexually active citizens (MSM) in the country.

Communicable diseases differ from other health threats or crises because they spread from person to person. Therefore, problems are often not restricted to 1 country. Various specific interventions are therefore justified by the difference in local systems, cultures, and situations. However, the principles of outbreak response are general, and countries can learn from each other. This study shows that countries have varying degrees of ability to respond quickly to an unexpected outbreak; these findings expose weaknesses in the outbreak control capacity in Europe. Although important steps have been taken for improvement (*14*), the quality of LGV outbreak control in Europe could benefit from uniform approaches in controlling other infectious diseases with potential for international spread and from exchanging information between countries.

The challenge for the future will be to coordinate outbreak management in various countries for which continuity and coherence are essential. This study shows that coordination should at least aim to provide guidance as to when and how alerts should be implemented by various countries as well as to establish uniform case definitions and ensure the availability of optimal diagnostic facil common strategies and that these should be developed with respect to treatment algorithms and contact tracing. Furthermore, quality systems following the whole chain of outbreak management (alert, outbreak control measures, implementation, and evaluation) are needed. These systems should be based on standard approaches to outbreak management followed by external review of implemented measures. More international collaboration is needed to improve response and to ensure high standards of practice in managing international outbreaks and threats caused by emerging or reemerging STIs.

Crisis situations increase the chance of overlooking essential steps in outbreak management because of time constraints, uncertainty, and the lack of substantial evidence in effective approaches to controlling new diseases. Furthermore, during outbreaks, key recommendations involve quick decision-making by professionals who often have no time for reevaluation. Although this need for quick decisions has been acknowledged for other threats like avian flu, SARS, or bioterrorism, little experience has been acquired with managing outbreaks of STIs. Our systematic approach could be helpful in preparing for or assessing the response to all kinds of public health emergencies.
